# Supplementary material for: Could Subtle Obstetrical Brachial Plexus Palsy Be Related to Unilateral B Glenoid Osteoarthritis?
Source: J Clin Med. 2021 Mar 12;10(6):1196. doi: 10.3390/jcm10061196 (PMC7999215; doi:10.3390/jcm10061196)
Supplement: Supplementary file 1 [file jcm-10-01196-s001.pdf]

Journal of
Clinical Medicine

MDPI

Supplementary Material

Table S1. Literature review of perinatal risk factors for OBPP development in the general population.

Author	Year	Journal	Population (*N*)	Incidence (%)
**Macrosomia (>4 kg)**				
Lalka et al.	2019	J Pediatr Orthop	966447	0.1%
Enomoto et al.	2016	PLoS One	97157	0.8%
Ganeshan et al.	2018	J Obstet Gynaecol India	88837	2.5%
Su et al.	2019	Ann Nutr Metab	73498	2.7%
Song et al.	2017	BMJ Open	1700976	3.1%
Zhao et al.	2020	PLoS One.	27289	3.1%
Ijäs et al.	2019	PLoS One	24577	3.2%
Tamez-Pérez et al.	2017	Endocrinol Diabetes Nutr	1189	5.1%
Soliman et al.	2018	Acta Biomed	12177	5.5%
Wang et al.	2020	BMC Pregnancy Childbirth	490143	6.0%
Liu et al.	2017	BMJ Open	213461	7.7%
Shang et al.	2020	Environ Sci Pollut Res Int	321521	7.8%
Mallouli et al.	2017	Tunis Med	10186	8.1%
Chen et al.	2020	Pediatr Obes	177841	8.2%
Davis et al.	2018	Am J Perinatol Rep	5937	9.7%
Litzky et al.	2018	Am J Obstet Gynecol	180184	9.8%
Yang et al.	2019	Diabetes Res Clin Pract	614175	11.2%
Kodan et al.	2020	PLoS One.	2019	12.8%
Wang et al.	2018	J Matern Fetal Neonatal Med	59424	13.9%
Moll et al.	2020	BMC Pregnancy Childbirth	4738	17.6%
Aberg et al.	2016	Acta Obstet Gynecol Scand	1030775	18.5%
**Fetal distress (hypoxia)***				
Coroneos et al.	2016	J neurosurg pediatr	2762996	0.4%
Andersson et al.	2016	Clinical Epidemiology	53022	0.6%
Lalka et al.	2019	J Pediatr Orthop	966447	1.1%
DeFrancesco et al.	2019	J Pediatr Orthop	3733760	1.9%
Schuit et al.	2016	J Matern Fetal Neonatal Med	241595	4.9%
Huang et al.	2020	Journal of Dermatology	2350330	5.0%
Hildén et al.	2019	Diabet Med	1455667	6.8%
Frohlich et al.	2020	Pediatrics International	1373612	7.2%
Stephansson et al.	2018	Plos one	798338	7.3%
Zhong et al.	2018	BMC Pregnancy Childbirth	23507597	14.3%
Heun-Johnson e al.	2019	J Perinatol	5475739	16.4%
**Gestational diabetes**				
Beyer et al.	2020	Eur Heart J	18151897	0.2%
Coroneos et al.	2016	J neurosurg pediatr	2762996	0.6%
Sorbye et al.	2020	BJOG	614432	0.7%
Olsen et al.	2017	Acta Obstet Gynecol Scand.	87792	0.9%
Barrett et al.	2020	PLoS Med	1924409	0.9%
Lalka et al.	2019	J Pediatr Orthop	966447	1.0%
Hildén et al.	2020	Diabet Med	1455667	1.0%
Leirgul et al.	2016	Obstet Gynecol.	914427	1.1%
Hildén et al.	2019	Diabet Med	1294006	1.1%
Yu et al.	2019	BMJ	2432000	1.1%
Fiaschi et al.	2016	Hum Reprod.	8093653	1.5%

Liu et al.	2018	Allergy	544677	1.5%
Freeman et al.	2017	Int J Gynecol Obstet	376325	1.8%
Holst et al.	2016	Fertil Steril.	372677	2.0%
Lehmann et al.	2019	Acta Obstet Gynecol Scand	57109	2.0%
Herrick et al.	2019	BMC Public Health	45810	2.4%
Strand-Holm et al.	2019	Midwifery	31297	2.5%
Nielsen et al.	2020	J Clin Endocrinol Metab	725482	2.7%
Cesta et al.	2019	BMJ Open Diabetes Res Care	5279231	2.8%
Sasaki et al.	2020	J Diabetes Investig	82972	2.8%
Jeppesen et al.	2017	Scand J Public Health.	566083	2.9%
Toijonen et al.	2019	Arch Gynecol Obstet	2056	3.3%
Artzi et al.	2020	Nat Med	451402	3.6%
Leeves et al.	2019	Tidsskr Nor Laegeforen	34915	3.8%
Kim et al.	2020	BMJ Open Diabetes Res Care	164053	4.0%
Papatheodorou et al.	2020	Sci Total Environ	1061937	4.1%
Schraw et al.	2020	Ann Epidemiol	6543385	4.1%
Yeung et al.	2017	J Diabetes Complications.	12036	4.2%
Kim et al.	2020	Diabetes Res Clin Pract	380208	4.5%
Balslev et al.	2019	Multiple sclerosis journal	27664	4.9%
Yang et al.	2019	Diabetes Res Clin Pract	650914	5.0%
Magtanong et al.	2019	J Matern Fetal Neonatal Med	12584918	5.1%
Baghlaf et al.	2019	J Matern Fetal Neonatal Med	7549763	5.2%
Yeung et al.	2017	J Diabetes Complications.	17912	5.8%
Farland et al.	2020	Cancer Causes Control	662630	5.9%
Robledo et al.	2017	Matern Child Health J.	4821	6.7%
Boyle et al.	2018	Plos one	96572	8.0%
Zeng et al.	2020	BMC Pregnancy Childbirth	237293	8.0%
Ijäs et al.	2019	Plos one	59057	9.6%
Kong et al.	2019	JAMA Pediatr	649043	15.2%
**Forceps delivery**				
DeFrancesco et al.	2019	J Pediatr Orthop	3733760	0.1%
Lalka et al.	2019	J Pediatr Orthop	966447	0.1%
Abdulrahman et al.	2019	Rev Bras Ginecol Obstet	5461	0.2%
Aberg et al.	2016	Acta Obstet Gynecol Scand	1332863	0.3%
Marschalek et al.	2018	Birth	222494	0.3%
Huebner et al.	2019	Arch Gynecol Obstet	27729	0.4%
Korbeľ et al.	2019	Ceska Gynekol		0.6%
Bailit et al.	2016	Am J Obstet Gynecol	2539	0.9%
Krizman et al.	2019	AJP Rep	73257	1.0%
Nembhard et al.	2019	Matern Child Health J	91622	1.4%
Domingues et al.	2016	Reprod Health	23894	1.5%
Larsen et al.	2016	Acta Obstet Gynecol Scand	2257724	2.1%
Biru et al.	2019	BMC Res Notes	14688	2.3%
Wesnes et al.	2017	Acta Obstet Gynecol Scand	7561	2.5%
Coroneos et al.	2016	J neurosurg pediatr	2762996	2.6%
**Vaginal breech delivery**				
Lalka et al.	2019	J Pediatr Orthop	966447	0.1%
Bin et al.	2017	Acta Obstet Gynecol Scand	1066599	0.1%
Hehir et al.	2018	Am J Obstet Gynecol	27044217	0.1%
Hinnenberg et al.	2019	Arch Gynecol Obstet	643340	0.1%
DeFrancesco et al.	2019	J Pediatr Orthop.	3733760	0.2%
Coroneos et al.	2016	J neurosurg pediatr	2762996	0.3%
Sucksdorff et al.	2018	Acta Paediatr	49533	0.3%
Waldenström et al.	2017	BMC Pregnancy Childbirth	436670	0.3%

Bergenhenegouwen et al.	2016	J Matern Fetal Neonatal Med	1543	0.4%
Pyykönen et al.	2017	Acta Obstet Gynecol Scand	3398586	0.4%
Macharey et al.	2017	BMC Pregnancy Childbirth	585581	0.5%
Macharey et al.	2018	J Perinat Med	415526	0.5%
Duffy et al.	2019	Acta Obstet Gynecol Scand	872224	0.7%
Benjamin et al.	2019	Birth Defects Res	1983	0.8%
Bjellmo et al.	2017	BMJ Open	520047	1.1%
Laine et al.	2020	Eur J Obstet Gynecol Reprod Biol	602095	1.4%
Wehberg et al.	2018	BMJ Open	206992	2.2%
Lindblad et al.	2020	BMC Pregnancy Childbirth	2129124	6.0%
**Shoulder dystocia**				
Ganeshan et al.	2018	J Obstet Gynaecol India	88837	0.1%
Heinonen et al.	2020	Acta Obstet Gynecol Scand	800484	0.2%
Gandhi et al.	2019	J Hand Surg Am	23385597	0.2%
Lalka et al.	2019	J Pediatr Orthop	966447	0.2%
Von Heideken et al.	2020	BMC Musculoskelet Disord	908324	0.2%
Kullinger et al.	2018	Sci Rep	1100049	0.2%
Lamminpää et al.	2016	Obes Res Clin Pract	249648	0.3%
Dahlberg et al.	2018	BMC Pregnancy Childbirth	11667	0.3%
DeFrancesco et al.	2019	J Pediatr Orthop	3733760	0.3%
Karahanoglu et al.	2016	Arch Gynecol Obstet	62268	0.5%
Turkmen et al.	2018	J Pregnancy	1373	0.7%
Larsen et al.	2016	Acta Obstet Gynecol Scand	2257724	0.8%
Freeman et al.	2017	Int J Gynecol Obstet	376325	1.0%
Ouzounian et al.	2016	J Reprod Med	13998	1.6%
Santos et al.	2018	JOGNN	19236	2.2%
Davis et al.	2018	Am J Perinatol Rep	5937	2.2%
Pillai et al.	2020	Birth	68966	2.6%
Coroneos et al.	2016	J neurosurg pediatr	2762996	2.8%
**Clavicular fracture**				
Rehm et al.	2019	J Obstet Gynaecol.	87461	0.1%
Coroneos et al.	2016	J neurosurg pediatr	2762996	0.2%
Gandhi et al.	2019	J Hand Surg Am.	23385597	0.2%
Kekki et al.	2020	Acta Paediatr	1203434	1.1%
Asena et al.	2020	J Neonatal Perinatal Med.	33480	2.3%
